# Pure Yolk sac presenting with inferior vena cava thrombus extending from bilateral external iliac veins to hepatic vein

**DOI:** 10.1590/S1677-5538.IBJU.2016.0142

**Published:** 2016

**Authors:** Oktay Ucer, Nalan Nese, Talha Muezzinoglu

**Affiliations:** 1 Department of Urology, Faculty of Medicine, Celal Bayar University, Manisa, Turkey; 2 Department of Pathology, Faculty of Medicine, Celal Bayar University, Manisa, Turkey

**Keywords:** Yolk Sac, Testicular Neoplasms, Vena Cava, Inferior, Thrombosis

## Abstract

**Introduction:**

Vena cava thrombus is an extremely rare complication of testicular tumors. We report on an unusual case of testicular tumor presenting with inferior vena cava thrombus extending from the left spermatic and bilateral external iliac veins to the hepatic vein.

**Case report:**

A-35-year old man presented with a 6-month history of left scrotal mass and a 1-day history of bilateral lower extremity edema. Computed tomography (CT) revealed the presence of thrombus extending from the left spermatic vein and bilateral external iliac veins to the hepatic vein, and multiple lymph node and lung metastases. 3 cycles of chemotherapy were given after the left high inguinal orchiectomy. Pathological examination demonstrated a pure yolk sac carcinoma with lymphovascular invasion and direct tumor extension into the left spermatic cord. CT and positron emission tompgraphy-CT obtained no findings of metastasis or recurrence at 3 months after the chemotherapy.

**Conclusion:**

We review this seldom case and discuss the literature with regard to its diagnosis and treatment.

## INTRODUCTION

Inferior vena cava thrombus due to a testicular tumor is an extremely rare condition. There have been a few reports of thrombus due to testicular tumor extended to inferior vena cava in literature. Most of pathological examinations revealed mixed germ cell carcinoma which generally consisted of embryonic carcinoma (1–3). We presented a case of pure yolk sac testicular tumor with the thrombus of inferior vena cava extended from the left spermatic and bilateral external iliac veins to the hepatic vein.

## CASE REPORT

**Figure 1:**
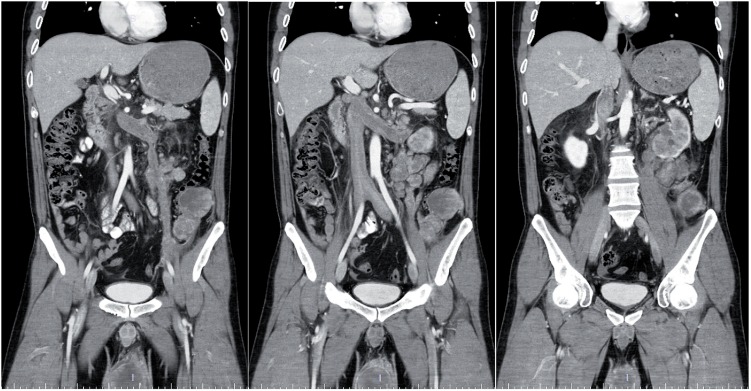
Computerized tomography scan of the abdomen demonstrating the inferior vena cava thrombus extended from the left spermatic vein and bilateral external iliac veins to the hepatic vein, retroperitoneal multiple metastasis and the impairment of the left renal function.

A 35-year-old man was admitted to the hospital complaining of bilateral lower extremity edema. He reported a six-month history of painless mass in the left scrotum. Physical examination disclosed a 10 × 6cm firm non-tender left scrotal mass. A thorax computed tomography (CT) demonstrated multiple metastases in both lungs. Abdomen enhanced CT revealed retroperitoneal multiple lymph node metastases, inferior vena cava thrombus extended from the left spermatic vein and bilateral external iliac veins to the hepatic vein and a decrease in the function of the left kidney due to a thrombus of the left renal vein (Figure-1). Color Doppler ultrasound confirmed complete occlusion of the inferior vena cava, the left renal and bilateral common iliac veins. Serum tumor markers were high except beta human chorionic gonadotropin (ß-HCG): lactate dehydrogenase (LDH), 441U/L (120-246); alpha-fetoprotein (AFP), >1000ng/mL (<8); ß-HCG, 0.2mIU/mL (<1.0).

**Figure 2:**
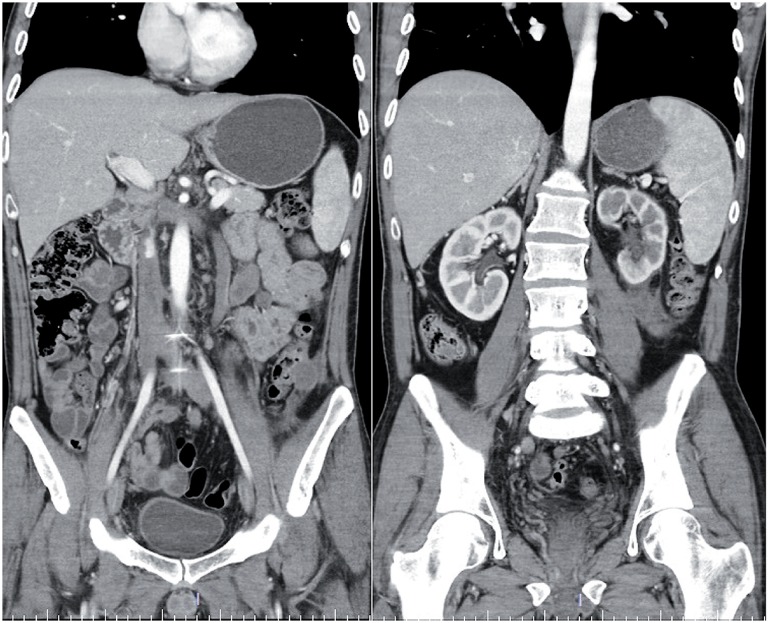
Computerized tomography scan of the abdomen demonstrating the inferior vena cava thrombus extended from the left spermatic vein and bilateral external iliac veins to the hepatic vein, no evidence of metastasis, and the improvement of the left renal function at 3 months after the treatment.

A left high inguinal orchiectomy was performed and pathological diagnosis was pure yolk sac carcinoma with lymphovascular invasion and direct tumor extension into the left spermatic cord. Three cycles of bleomycin, etoposide, and cisplatin (BEP) were given. A nearly complete response in the retroperitoneal and lung metastases was observed by thorax and abdominal CT (Figure-2), tumor markers were normalized at 3 months after this chemotherapy. Although the patient did not have any several respiratory complaints, thorax CT showed partial embolism in the right pulmonary artery and bronchial mass. Anticoagulant treatment was begun and then bronchoscopy with biopsy was performed. Pathological and microbiological examination revealed mycobacterium tuberculosis. Anti-tuberculosis therapy was started and an inferior vena cava filter was implanted through the right internal jugular vein. The inferior vena cava remained occluded, with evidence of collateralization such as the improvement of the left renal function. CT and positron emission tomography (PET-CT) showed no findings of metastasis or local recurrence at 6 months after the surgery.

## DISCUSSION

Thrombus of inferior vena cava due to testis tumor is an extremely seldom condition. In earlier cases (1–7), most of pathological examinations revealed embryonic carcinoma or mixed non-seminomatous germ cell carcinoma that usually consisted of embryonic carcinoma. Kinebuchi et al. reported that the embryonic carcinoma component in the primary testicular tumor with inferior vena cava thrombosis was relatively high (7). This high rate may be due to the aggressive behavior of embryonic carcinoma. The presented case was the first patient who had inferior vena cava thrombus due to pure yolk sac testis tumor according to our knowledge of the literature. Although yolk sac is a less aggressive tumor than embryonic carcinoma, it caused the inferior vena cava thrombosis of the present case. This may be due to the long term history (six months) of painless testicular mass in the patient.

Since the inferior vena cava thrombus lengths of earlier cases had been generally short, these patients were treated with either chemotherapy alone or surgery excision of thrombus after the chemotherapy (4–7). Only one case was reported with inferior vena cava thrombus extended from the infrarenal level to the common iliac veins (8). This patient underwent high inguinal orchiectomy and chemotherapy. Since the radiological imaging showed no evidence of metastasis at 4 months the treatment, additional therapy was not performed for this case. Surgery excision was not performed for the occluded inferior vena cava because of collateralization.

Our case had inferior vena cava thrombus extended from the left spermatic vein and bilateral external iliac veins to the hepatic vein. The radiological imaging (CT and PET-CT) indicated no evidence of metastasis or recurrence after orchiectomy and chemotherapy. Although the inferior vena cava remained occluded completely, there were evidences of collateralization such as the improvement of the left renal function and disappearance of lower extremity edema. Overall, we discussed with the present patient and decided not to performed surgery excision of inferior vena cava thrombus.

Testicular tumor presenting with thrombosis of the inferior vena cava, with or without subsequent pulmonary embolism, can be managed with a combination of anticoagulation and/ or inferior vena cava filter placement. The present patient was begun anticoagulation therapy before the chemotherapy for the risk of pulmonary embolism but a filter was not placed into the inferior vena cava. After partial pulmonary embolism occurred, the filter was placed into inferior vena cava. We suggest that a filter should be placed and started anticoagulation therapy before the chemotherapy because of the risk of pulmonary embolism.
